# Three-Dimensional ECM-Functionalized PAN/C500 Nanofiber Scaffolds Induce Cytoskeletal Remodeling and Stemness-Associated Molecular Changes in Glioblastoma Cells

**DOI:** 10.3390/biom16081077

**Published:** 2026-07-23

**Authors:** Ihsan Nalkiran, Hatice Sevim Nalkiran, Derya Bal Altuntas, Atilla Eren Mamuk, Cagdas Kocak, Ebiha Can, Sema Aslan

**Affiliations:** 1 Department of Medical Biology, Faculty of Medicine, Recep Tayyip Erdogan University, Rize 53100, Türkiye; ihsan.nalkiran@erdogan.edu.tr (I.N.); ebiha_can22@erdogan.edu.tr (E.C.); 2 Department of Bioengineering, Faculty of Engineering and Architecture, Recep Tayyip Erdogan University, Rize 53100, Türkiye; derya.balaltuntas@erdogan.edu.tr; 3 Department of Physics, Faculty of Science, Mugla Sitki Kocman University, Muğla 48100, Türkiye; aemamuk@mu.edu.tr (A.E.M.); cagdaskocak@mu.edu.tr (C.K.); 4 Department of Chemistry, Faculty of Science, Mugla Sitki Kocman University, Muğla 48100, Türkiye; semaaslan@mu.edu.tr

**Keywords:** glioblastoma, three-dimensional cell culture, polyacrylonitrile nanofibers, ECM functionalization, cytoskeletal remodeling, stemness

## Abstract

Glioblastoma (GBM) is the most aggressive primary brain tumor and remains associated with poor clinical outcomes despite advances in surgical and adjuvant therapies. The tumor microenvironment, particularly extracellular matrix (ECM) interactions, plays a crucial role in regulating glioblastoma progression, cellular plasticity, and therapeutic resistance. Therefore, physiologically relevant three-dimensional (3D) models are needed to better recapitulate GBM biology. In this study, we investigated the effects of ECM-functionalized polyacrylonitrile/coumarin-500 (PAN/C500) nanofiber scaffolds on the phenotype of LN-18 and U-87 MG glioblastoma cells cultured under 3D conditions. Cytoskeletal organization was assessed by phalloidin staining and live-cell vimentin imaging, while epithelial–mesenchymal transition (EMT)-associated proteins and stemness-related markers were analyzed by Western blotting. ECM-functionalized 3D PAN/C500 scaffolds promoted significant cytoskeletal remodeling, altered EMT-associated protein expression, and increased the expression of stemness-associated proteins, particularly SOX2, NANOG, and Nestin, compared with conventional 2D cultures. These responses were accompanied by cell line-dependent phenotypic adaptations, indicating that the engineered microenvironment influences glioblastoma cell behavior. This platform may serve as a valuable model for investigating glioblastoma biology and microenvironment-associated molecular adaptations in vitro.

## 1. Introduction

Glioblastoma (GBM) is the most common and aggressive primary malignant brain tumor in adults and remains associated with poor clinical outcomes despite significant advances in surgical resection, radiotherapy, and chemotherapy. The median survival of patients with GBM remains approximately 12–15 months, while the five-year survival rate is below 10% [1,2]. The remarkable aggressiveness of GBM is largely attributed to extensive intratumoral heterogeneity, high cellular plasticity, diffuse invasion into surrounding brain tissue, and the development of therapeutic resistance [3,4].

The biological behavior of GBM is strongly influenced by the tumor microenvironment (TME), which consists of extracellular matrix (ECM) components, soluble signaling molecules, hypoxic niches, and interactions with surrounding stromal and neural cells [5,6]. Compared with normal brain tissue, glioblastoma-associated ECM exhibits substantial compositional and architectural alterations, including increased deposition of fibronectin, collagen, and other matrix proteins that facilitate tumor progression and invasion [7,8].

Among the cellular processes influenced by the TME, phenotypic plasticity and stemness have emerged as critical determinants of glioblastoma progression. Glioblastomas contain subpopulations of glioma stem-like cells that contribute to tumor initiation, recurrence, therapeutic resistance, and disease progression [9]. These cells are characterized by the expression of stemness-associated transcription factors, including SOX2, NANOG, OCT4, and c-MYC, which regulate self-renewal, cellular reprogramming, and maintenance of undifferentiated states [10,11,12]. In particular, SOX2 has been identified as a key regulator of glioma stem cell maintenance, while NANOG and OCT4 participate in transcriptional networks associated with pluripotency and tumor cell plasticity [13,14,15,16].

In addition to stemness-related pathways, increasing evidence suggests that interactions between tumor cells and the surrounding ECM influence cytoskeletal organization and mesenchymal adaptation [17]. Changes in actin and intermediate filament dynamics, particularly vimentin expression, have been associated with enhanced migratory capacity, invasive behavior, and adaptation to complex microenvironments in malignant tumors [18,19,20]. These observations suggest that physiologically relevant tumor models should ideally reproduce both the structural and biochemical characteristics of the native microenvironment.

Traditional two-dimensional (2D) cell culture systems remain widely used because of their simplicity and reproducibility; however, they fail to accurately recapitulate the spatial architecture, mechanical properties, and cell–ECM interactions present in tumors. Consequently, cellular responses observed in monolayer cultures frequently differ from those observed in vivo, limiting their translational relevance for therapeutic development [21,22]. Three-dimensional (3D) culture systems have therefore emerged as more physiologically relevant alternatives capable of reproducing key features of the tumor microenvironment, including cell–cell interactions, ECM signaling, oxygen gradients, and stem cell niches [23,24,25,26].

Among the available 3D culture platforms, electrospun nanofiber scaffolds have attracted considerable interest because their highly porous architecture closely resembles the structural organization of native ECM. These scaffolds support cell adhesion, migration, proliferation, and tissue-like cellular organization while allowing incorporation of biologically relevant ECM components [27,28]. Furthermore, recent advances in nanofiber-based biomaterials have enabled the development of multifunctional tumor models that provide structural support and biologically relevant microenvironments for cancer cell culture [29,30].

In our previous studies, we systematically developed, optimized, and comprehensively characterized a PAN/C500 nanofiber platform for glioblastoma cell culture applications, including comparisons of different PAN-based scaffold formulations, physicochemical characterization, electrochemical performance, biological compatibility, and systematic optimization of cell line-specific ECM coating conditions for LN-18 and U-87 MG cells. Previous cross-sectional H&E analyses also demonstrated cellular infiltration of both LN-18 and U-87 MG cells into the nanofiber layer [31,32]. Building upon these findings, the present study was designed to biologically characterize the previously optimized ECM-functionalized PAN/C500 scaffold rather than to investigate the individual contributions of its constituent materials. We aimed to investigate how ECM-functionalized PAN/C500 scaffolds influence glioblastoma cell phenotype under 3D culture conditions. To our knowledge, the effects of ECM-optimized PAN/C500 scaffolds on EMT-associated proteins, cytoskeletal organization, and stemness-related characteristics of glioblastoma cells have not been previously explored. Specifically, we evaluated cytoskeletal organization, vimentin distribution, EMT-associated protein expression, and stemness-related markers in LN-18 and U-87 MG glioblastoma cells cultured under conventional 2D and biomimetic 3D conditions. We hypothesized that ECM-supported 3D scaffolds would promote phenotypic adaptations associated with cytoskeletal remodeling, cellular plasticity, and stemness-associated molecular changes, thereby generating a more physiologically relevant in vitro glioblastoma model.

## 2. Materials and Methods

### 2.1. Fabrication of PAN/C500 Nanofiber-Coated Electrodes

Electrospun polyacrylonitrile (PAN) nanofibers integrated with coumarin-500 (C500) were deposited onto indium tin oxide (ITO) substrates to generate electroactive 3D scaffolds. The fabrication procedure, including solution preparation, electrospinning parameters, and post-processing steps, was performed as previously described in our earlier studies [31,32]. Briefly, PAN-based nanofibers were electrospun under optimized conditions to obtain uniform fiber morphology and stable coating on ITO surfaces. The incorporation of C500 was carried out to enhance the electroactive properties of the scaffold.

### 2.2. Surface Functionalization with Extracellular Matrix Components

To promote cell adhesion and proliferation, nanofiber-coated substrates were functionalized with ECM proteins. Based on our previous optimization experiments on the PAN/C500 platform, fibronectin (1 µg/mL) (Cat no:sc-29011, Santa Cruz Biotechnology Inc., Dallas, TX, USA) and collagen IV (10 µg/mL) (Collagen from human placenta Bornstein and Traub Type IV, Cat no: C5533, Saint Louis, MO, USA) were selected as the optimal ECM coatings for LN-18 and U-87 MG cells, respectively, and were therefore used throughout the present study [32]. ECM coating was performed by incubating the substrates with the respective protein solutions under sterile conditions prior to cell seeding.

### 2.3. Cell Culture Conditions

The human glioblastoma cell line LN-18 was obtained from the American Type Culture Collection (ATCC, Manassas, VA, USA). The human glioblastoma-like cell line U-87 MG was kindly provided by Prof. Sevgi Irtegun Kandemir (Department of Medical Biology, Dicle University, Diyarbakır, Türkiye). Both cell lines were maintained in laboratory stocks and used throughout the study. The cell lines were cultured in Dulbecco’s Modified Eagle Medium (DMEM) (Cat. No. 41966-029, Gibco, Life Technologies Limited, Paisley, UK) supplemented with 10% fetal bovine serum (FBS) (Cat. No. FBS-HI-11A, Capricorn Scientific, Ebsdorfergrund, Germany) and standard antibiotics under humidified conditions at 37 °C with 5% CO_2_. Both LN-18 and U-87 MG cell lines were routinely screened for mycoplasma contamination using the ABMgood Mycoplasma PCR Detection Kit (Cat. No. G238, ABMgood, Richmond, BC, Canada), and all cultures used in this study were confirmed to be mycoplasma-free.

### 2.4. 2D and 3D Cell Seeding on PAN/C500 Scaffolds

PAN/C500 scaffolds were selected based on their previously demonstrated ability to support glioblastoma cell growth and scaffold colonization.

To compare conventional and biomimetic culture conditions, cells were cultured on both 2D surfaces and 3D ITO/PAN/C500 scaffolds. Based on our previous ECM optimization study [32], fibronectin (1 µg/mL) and collagen IV (10 µg/mL) were selected as the optimal coatings for LN-18 and U-87 MG cells, respectively. Accordingly, all required surfaces were coated with the corresponding ECM component prior to cell seeding. Subsequently, 1.25 × 10^5^ cells were seeded into each well of a 12-well plate containing either 2D culture surfaces or 3D PAN/C500 scaffolds and maintained for 7 days under standard culture conditions (37 °C, 5% CO_2_). Cells were cultured in DMEM supplemented with 10% FBS and 1% penicillin–streptomycin. The culture medium was replaced every 48 h throughout the experimental period. Following the 7-day culture period, samples were processed for fluorescence imaging and Western blot analyses to evaluate cytoskeletal organization, EMT-associated protein expression, and stemness-related characteristics under 2D and 3D culture conditions.

### 2.5. Phalloidin Staining for Visualization of Actin Filaments

After 7 days of incubation, cells were stained with phalloidin to visualize actin filaments (F-actin) using the Phalloidin iFluor 647 reagent (Catalog no: AB176759, Abcam, Waltham, MA, USA). A stock solution was prepared by dissolving the reagent in 30 µL of DMSO and stored as 1 µL aliquots at −20 °C. For working solutions, the stock was diluted in PBS at a 1:1000 ratio, in accordance with the manufacturer’s instructions. Fixation, permeabilization, and staining procedures were performed following the manufacturer’s protocol. Finally, nuclei were counterstained with Hoechst dye, and fluorescence imaging was carried out using a fluorescence microscope (Zeiss Axio Imager, Oberkochen, Germany). Fluorescence images were acquired using identical acquisition settings, with a consistent focal plane maintained for all quantitative analyses.

### 2.6. Live-Cell Staining for Visualization of Vimentin Protein

Cells cultured on both 2D and 3D surfaces for 7 days were stained to visualize vimentin, a cytoplasmic intermediate filament protein and key component of the cytoskeleton, using BioTracker TiY Vimentin Live Cell Dye (EMD Millipore, SCT059, Darmstadt, Germany). A stock solution of the dye was prepared in DMSO, and the working solution was diluted in DMEM supplemented with 10% FBS to achieve a final concentration of 1 µM. The staining solution was added directly to the cells, which were then incubated at 37 °C for 60 min under standard culture conditions. Following incubation, cells were washed with PBS to remove excess dye. Nuclei were counterstained with Hoechst dye, and fluorescence imaging was performed using a fluorescence microscope (Zeiss Axio Imager, Oberkochen, Germany). Images were acquired under identical imaging settings, and the focal plane was kept consistent for quantitative comparisons.

### 2.7. Fluorescence Quantification

Fluorescence quantification was performed using ImageJ software (Version 1.54g; National Institutes of Health, Bethesda, MD, USA). Images were acquired from three independent biological experiments, and three randomly selected non-overlapping fields were captured at 40× magnification for each experiment. For vimentin analysis, individual cells were manually outlined as regions of interest (ROIs), and Corrected Total Cell Fluorescence (CTCF) was calculated as Integrated Density − (Area × Mean Background Fluorescence), where the background fluorescence was determined from cell-free regions within each image. For F-actin analysis, fluorescence was quantified on a field basis using the same CTCF approach. Cell-normalized fluorescence intensity was subsequently calculated by dividing the field CTCF by the number of Hoechst-positive nuclei counted using the Cell Counter plugin in ImageJ. The mean value obtained from the three fields represented one biological replicate (*n* = 3).

### 2.8. Western Blotting

Cells were lysed using RIPA buffer containing protease inhibitors and centrifuged at 14,000 rpm for 15 min at 4 °C. Protein concentrations were determined using the Pierce Dilution-Free Rapid Gold BCA Protein Assay Kit (Thermo Fisher Scientific, Waltham, MA, USA; Cat. No. A55861). Equal amounts of protein (25 μg) were denatured with 2× Laemmli Sample Buffer (Ecotech Biotechnology, Erzurum, Türkiye; Cat. No. LSB-2x), separated on 10% SDS-PAGE gels, and transferred onto PVDF membranes (GVS North America, Sanford, ME, USA; Cat. No. 1212639).

Membranes were incubated overnight at 4 °C with primary antibodies against SOX2 (Elabscience; Cat. No. E-AB-18159, Houston, Texas, USA), OCT4 (Thermo Fisher Scientific; Cat. No. PA5-27438, Waltham, MA USA), NANOG (Proteintech; Cat. No. 14295-1-AP, Rosemont, IL, USA), Nestin (Elabscience; Cat. No. E-AB-63599, Houston, TX, USA), E-cadherin (Affinity Biosciences; Cat. No. AF0131, Cincinnati, OH, USA), N-cadherin (Abcam; Cat. No. ab76011, Cambridge, UK), Vimentin (Abcam; Cat. No. ab92547, Cambridge, UK), β-actin (Proteintech; Cat. No. 20536-1-AP), and GAPDH (Abclonal Technology; Cat. No. AC033, Woburn, MA, USA). After incubation with HRP-conjugated secondary antibodies, Anti-Mouse IgG (HRP) (Cell Signaling Technology, Danvers, MA, USA; Cat. No. 7076S) and Goat Anti-Rabbit IgG H&L (HRP) (Abcam, Cambridge, UK; Cat. No. ab205718), protein bands were visualized using Clarity Western ECL Substrate (Bio-Rad Laboratories, Hercules, CA, USA; Cat. No. 1705060) and imaged with a ChemiDoc Imaging System (Bio-Rad Laboratories, Hercules, CA, USA). Band intensities were quantified using ImageJ software (National Institutes of Health, Bethesda, MD, USA). Densitometric values were normalized to the corresponding housekeeping protein (GAPDH or β-actin) detected on the same membrane, and the normalized values were used for statistical analysis. All experiments were independently repeated three times.

### 2.9. Statistical Analysis

Fluorescence imaging data were analyzed using the GraphPad QuickCalcs online *t*-test calculator (2026 GraphPad Software, San Diego, CA, USA; https://www.graphpad.com/quickcalcs/ttest1/; accessed on 13 May 2026), and comparisons between 2D and 3D culture conditions were performed using an unpaired two-tailed Student’s *t*-test. Western blot densitometric data were analyzed using IBM SPSS Statistics software (version 25; IBM Corp., Armonk, NY, USA). Statistical significance among multiple experimental groups was determined by one-way analysis of variance (ANOVA) followed by Tukey’s multiple comparison test. All quantitative experiments were performed using three independent biological replicates (*n* = 3). Data are presented as mean ± standard deviation (SD). Differences were considered statistically significant at *p* < 0.05.

## 3. Results

### 3.1. EMT-Associated Protein Expression in Glioblastoma Cells Cultured Under 2D and 3D Conditions

**Figure 1:**
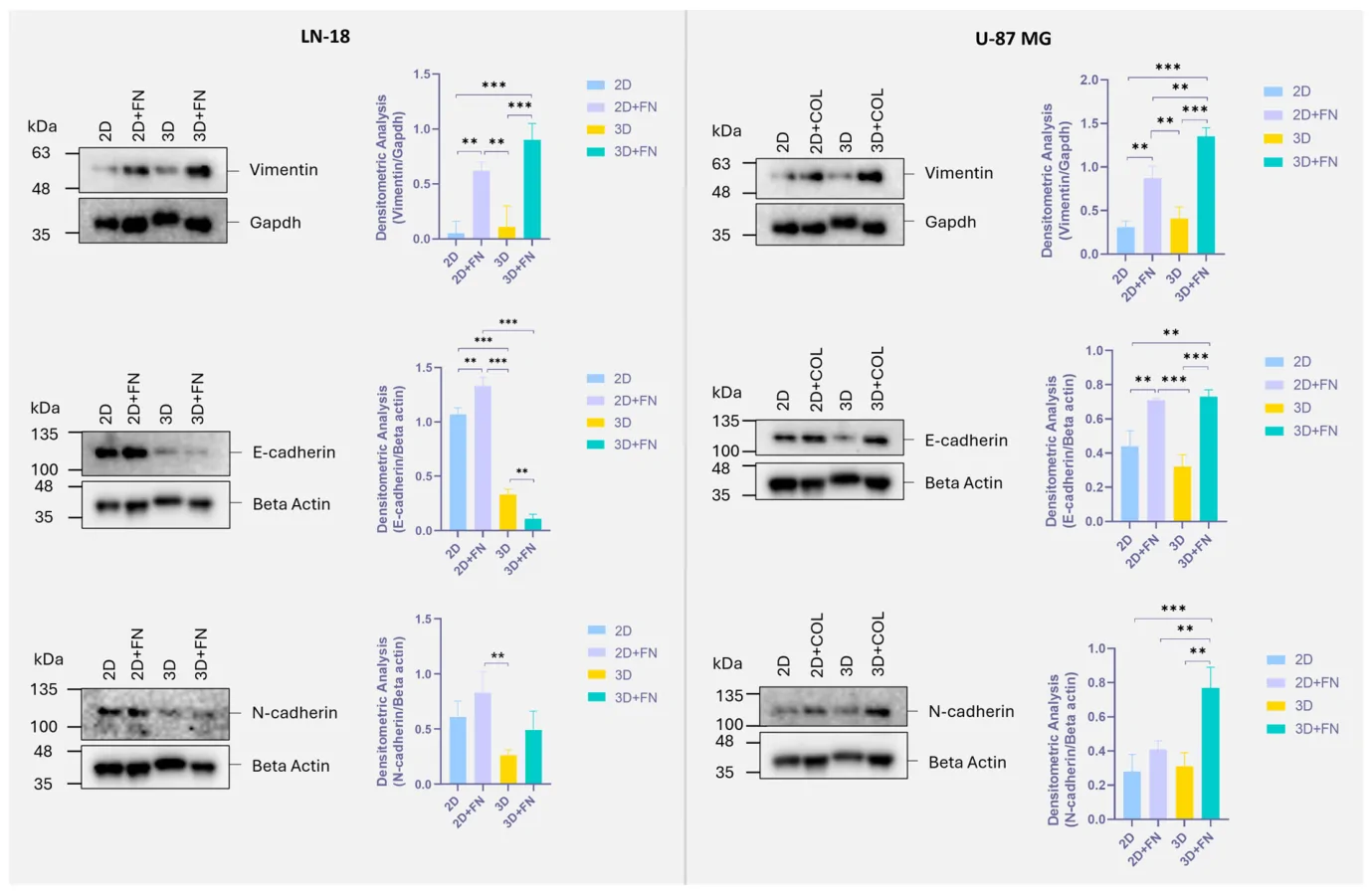
Expression of EMT-associated proteins in LN-18 and U-87 MG glioblastoma cells cultured under 2D and 3D conditions. Representative Western blot images and densitometric analyses of vimentin, E-cadherin, and N-cadherin expression in LN-18 and U-87 MG cells cultured on conventional 2D surfaces and 3D ITO/PAN/C500 scaffolds for 7 days. LN-18 cultures were maintained in the presence or absence of fibronectin (FN), whereas U-87 MG cultures were maintained in the presence or absence of collagen IV (COL). Protein expression levels were normalized to GAPDH or β-actin as indicated. For U-87 MG cells, the β-actin loading control shown for E-cadherin is shared with Nestin (Figure 4), as both proteins were detected sequentially on the same membrane following stripping and re-probing. Data are presented as mean ± SD from three independent experiments (*n* = 3). Statistical significance was determined using one-way ANOVA followed by Tukey’s multiple comparison test. *p* < 0.01 (**), and *p* < 0.001 (***). Original uncropped Western blot images are provided in Supplementary Figures S1 and S2.

Western blot analysis revealed culture condition-dependent changes in the expression of EMT-associated proteins, including vimentin, E-cadherin, and N-cadherin, in both LN-18 and U-87 MG glioblastoma cells (Figure 1).

In LN-18 cells, vimentin expression was significantly increased in the 2D+FN group compared with 2D cultures without fibronectin coating (*p* < 0.01), indicating that fibronectin alone was sufficient to promote mesenchymal marker expression. The highest vimentin expression was observed in the 3D+FN group, which was significantly elevated compared with both 3D cultures without fibronectin (*p* < 0.001) and conventional 2D cultures (*p* < 0.001). In contrast, cells cultured on 3D scaffolds without fibronectin exhibited only minimal vimentin expression.

Analysis of E-cadherin expression in LN-18 cells revealed a markedly different pattern. E-cadherin levels remained high under 2D and 2D+FN conditions but were significantly reduced following culture on 3D scaffolds. The lowest expression was detected in the 3D+FN group, which showed significantly lower E-cadherin levels than both 2D and 2D+FN cultures (*p* < 0.001). Furthermore, E-cadherin expression was significantly lower in 3D+FN cultures than in 3D cultures without fibronectin (*p* < 0.01). N-cadherin expression showed more moderate alterations, with the highest level observed in the 2D+FN group. Although N-cadherin expression decreased in cells cultured on 3D scaffolds, a partial recovery was observed in the 3D+FN group. Overall, the combination of increased vimentin and reduced E-cadherin expression suggests a shift toward a more mesenchymal-like phenotype in LN-18 cells under 3D ECM-supported culture conditions.

In U-87 MG cells, vimentin expression followed a similar trend. Both ECM supplementation and 3D culture increased vimentin levels, with the highest expression observed in the 3D+COL group. Vimentin expression in this group was significantly higher than in 2D (*p* < 0.001), 2D+COL (*p* < 0.01), and 3D cultures without collagen (*p* < 0.001). E-cadherin expression was also significantly elevated in the 2D+COL and 3D+COL groups compared with the corresponding cultures without collagen IV coating (*p* < 0.01), indicating that collagen IV strongly influenced epithelial marker expression in this cell line. In parallel, N-cadherin expression was markedly increased in the 3D+COL group and was significantly higher than in all other experimental groups (*p* < 0.01–0.001).

These findings demonstrate that ECM-functionalized 3D PAN/C500 scaffolds induce substantial alterations in EMT-associated protein expression in both glioblastoma cell lines. While LN-18 cells exhibited increased vimentin expression and loss of E-cadherin, U-87 MG cells showed simultaneous upregulation of both epithelial and mesenchymal-like molecular markers, suggesting a cell line-dependent phenotypic adaptation to the 3D microenvironment.

### 3.2. Vimentin Expression and Distribution in 2D and 3D Cultures

**Figure 2:**
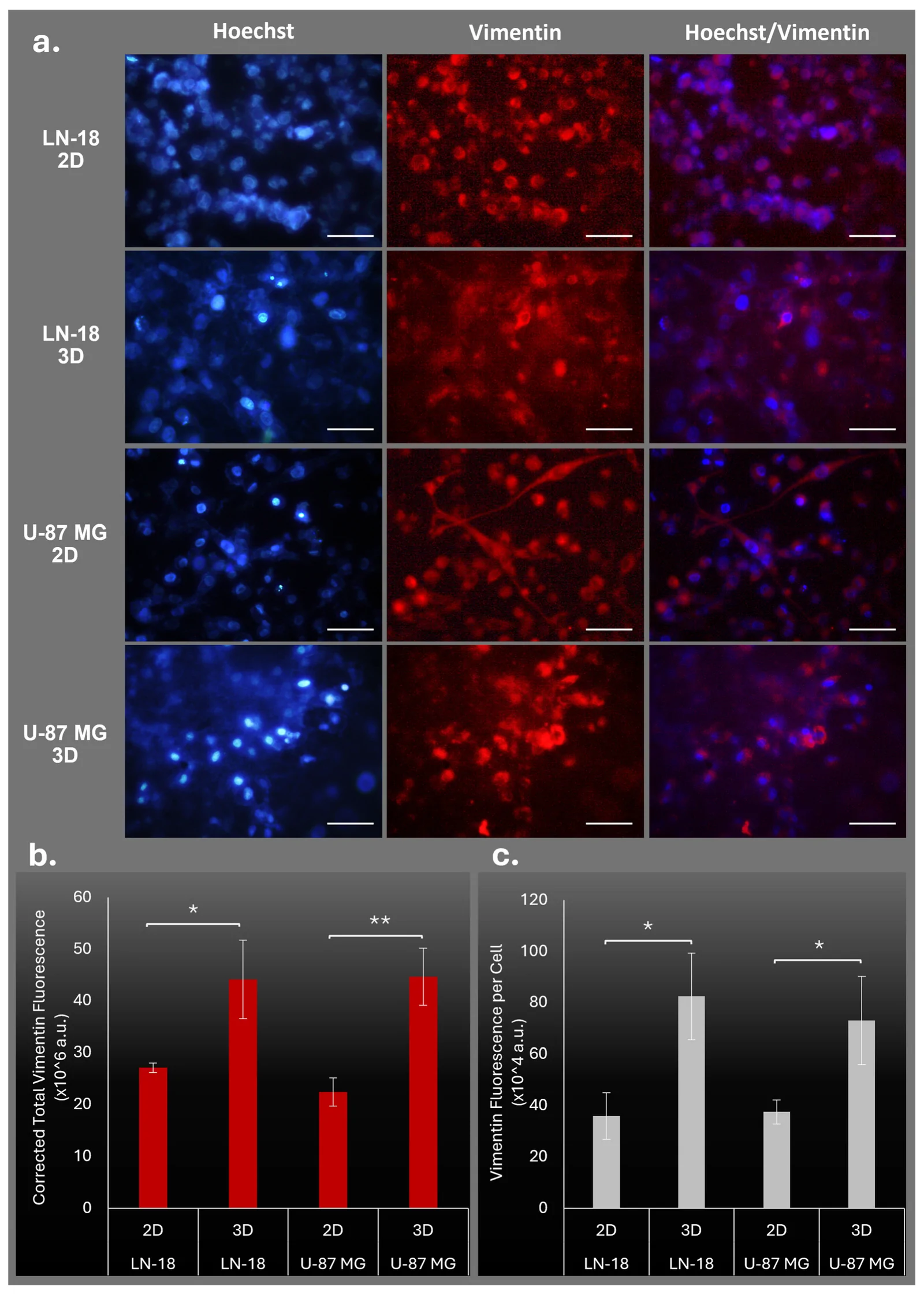
Vimentin expression and quantitative analysis in LN-18 and U-87 MG cells cultured under 2D and 3D conditions: (**a**) Representative fluorescence images of LN-18 and U-87 MG glioblastoma cells cultured on 2D surfaces and 3D ITO/PAN/C500 scaffolds for 7 days. Nuclei are stained with Hoechst (blue), and vimentin is visualized using a live-cell vimentin dye (red). Merged images (Hoechst/Vimentin) demonstrate differences in vimentin distribution and intensity between culture conditions. Images were acquired at 40× magnification. Scale bars: 50 µm. (**b**) Quantification of corrected total vimentin fluorescence intensity in LN-18 and U-87 MG cells under 2D and 3D conditions. (**c**) Quantification of vimentin fluorescence intensity normalized per cell. Data are presented as mean ± standard deviation (SD). Statistical significance was determined using Student’s *t*-test. *p* < 0.05 (*), *p* < 0.01 (**).

Live-cell fluorescence imaging revealed marked differences in vimentin distribution between 2D and 3D culture conditions in both LN-18 and U-87 MG glioblastoma cells (Figure 2). In LN-18 cells, vimentin staining was relatively weak and diffusely distributed under 2D conditions, whereas cells cultured on 3D PAN/C500 scaffolds exhibited a stronger, more organized vimentin network with increased fluorescence intensity (Figure 2a). Similarly, U-87 MG cells cultured under 3D conditions exhibited enhanced vimentin staining and more prominent filamentous structures compared with their 2D counterparts.

Quantitative analysis of corrected total vimentin fluorescence demonstrated a significant increase in LN-18 cells cultured on 3D scaffolds compared with 2D cultures (*p* < 0.05) (Figure 2b). A comparable trend was observed in U-87 MG cells, where total vimentin fluorescence was significantly elevated under 3D conditions (*p* < 0.01). When fluorescence values were normalized on a per-cell basis, vimentin intensity remained significantly higher in 3D cultures for both LN-18 and U-87 MG cells (*p* < 0.05) (Figure 2c). These findings indicate that the increased vimentin signal observed in 3D cultures is not solely attributable to differences in cell density, but rather reflects enhanced vimentin expression and reorganization at the cellular level.

The fluorescence imaging results were consistent with the Western blot findings presented in Figure 1, further supporting the notion that culture on 3D PAN/C500 scaffolds promotes vimentin-associated cytoskeletal remodeling in glioblastoma cells.

### 3.3. Cytoskeletal Organization and F-Actin Distribution in 2D and 3D Cultures

**Figure 3:**
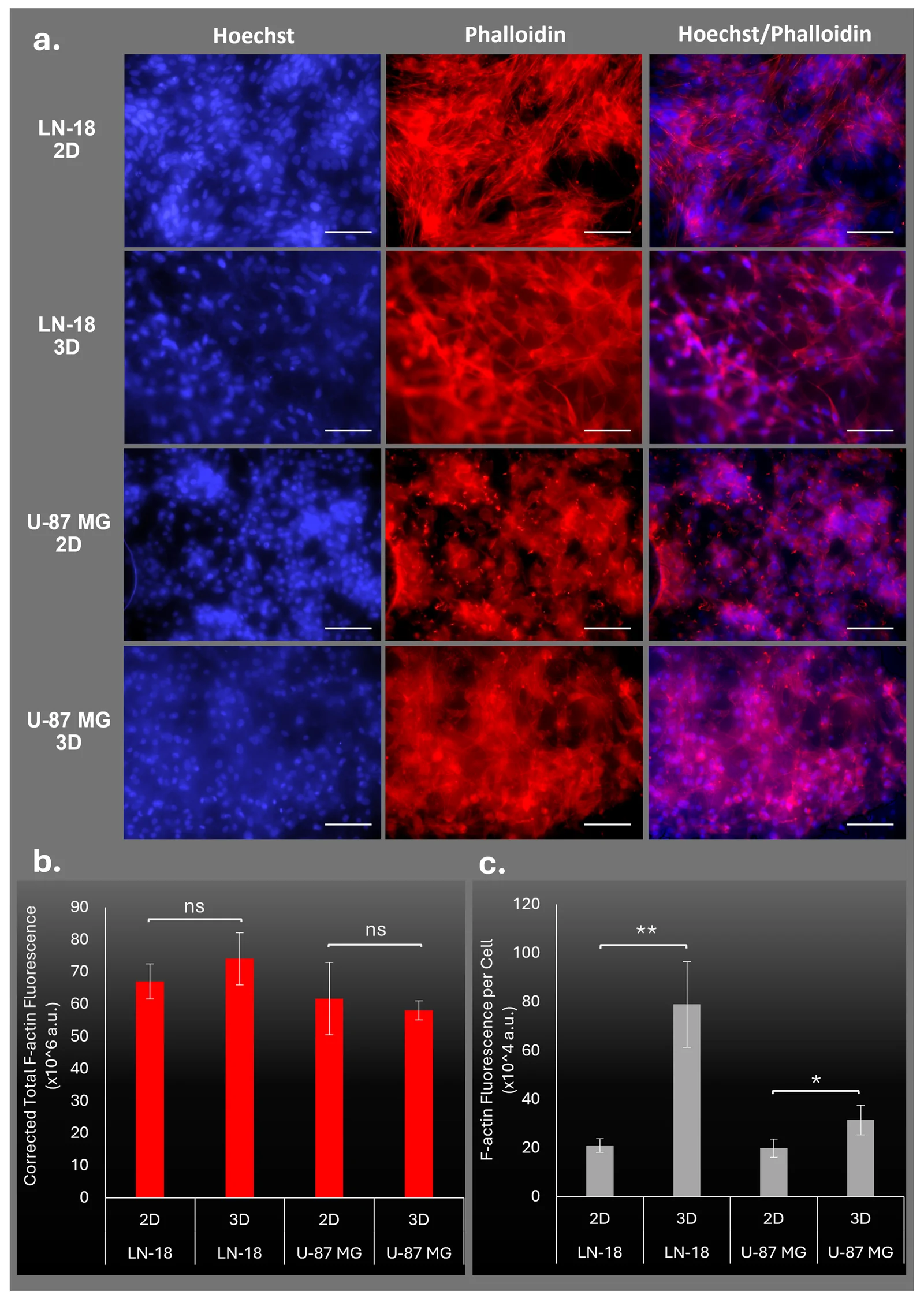
Cytoskeletal organization and quantitative analysis of F-actin in LN-18 and U-87 MG cells cultured under 2D and 3D conditions: (**a**) Representative fluorescence images of LN-18 and U-87 MG glioblastoma cells cultured on 2D surfaces and 3D ITO/PAN/C500 scaffolds for 7 days. Nuclei are stained with Hoechst (blue), and F-actin filaments are visualized using phalloidin (red). Merged images (Hoechst/Phalloidin) highlight differences in cytoskeletal organization between 2D and 3D culture conditions. Images were acquired at 40× magnification. Scale bars: 50 µm. (**b**) Quantification of corrected total F-actin fluorescence intensity in LN-18 and U-87 MG cells under 2D and 3D conditions. (**c**) Quantification of F-actin fluorescence intensity normalized per cell. Data are presented as mean ± standard deviation (SD). Statistical significance was determined using Student’s *t*-test. *p* < 0.05 (*), *p* < 0.01 (**), and ns: non-significant.

Phalloidin staining was performed to evaluate the impact of 3D culture conditions on actin cytoskeletal organization in glioblastoma cells (Figure 3). Representative fluorescence images demonstrated distinct differences in F-actin architecture between 2D and 3D cultures in both LN-18 and U-87 MG cells. In LN-18 cells, F-actin filaments appeared more organized and interconnected under 3D conditions, forming a dense filamentous network throughout the scaffold surface (Figure 3a). Similarly, U-87 MG cells cultured on 3D PAN/C500 scaffolds exhibited a more extensive and spatially distributed actin network compared with cells maintained under conventional 2D conditions.

Quantitative analysis of corrected total F-actin fluorescence revealed no significant differences between 2D and 3D cultures in either LN-18 or U-87 MG cells (Figure 3b), indicating that overall actin content remained relatively stable across culture conditions. However, normalization of fluorescence intensity to cell number revealed significant alterations in cytoskeletal organization. In LN-18 cells, F-actin fluorescence per cell was significantly increased in 3D cultures compared with 2D cultures (*p* < 0.01) (Figure 3c). Likewise, U-87 MG cells exhibited a significant increase in F-actin intensity per cell under 3D conditions (*p* < 0.05).

The absence of changes in total F-actin fluorescence, together with the significant increase in cell-normalized fluorescence, suggests that 3D culture primarily affects actin organization and distribution rather than total actin abundance. These findings indicate enhanced cytoskeletal remodeling in response to the 3D microenvironment and are consistent with the increased vimentin expression observed in Figure 1 and Figure 2.

### 3.4. Stemness-Associated Protein Expression in Glioblastoma Cells Cultured Under 2D and 3D Conditions

**Figure 4:**
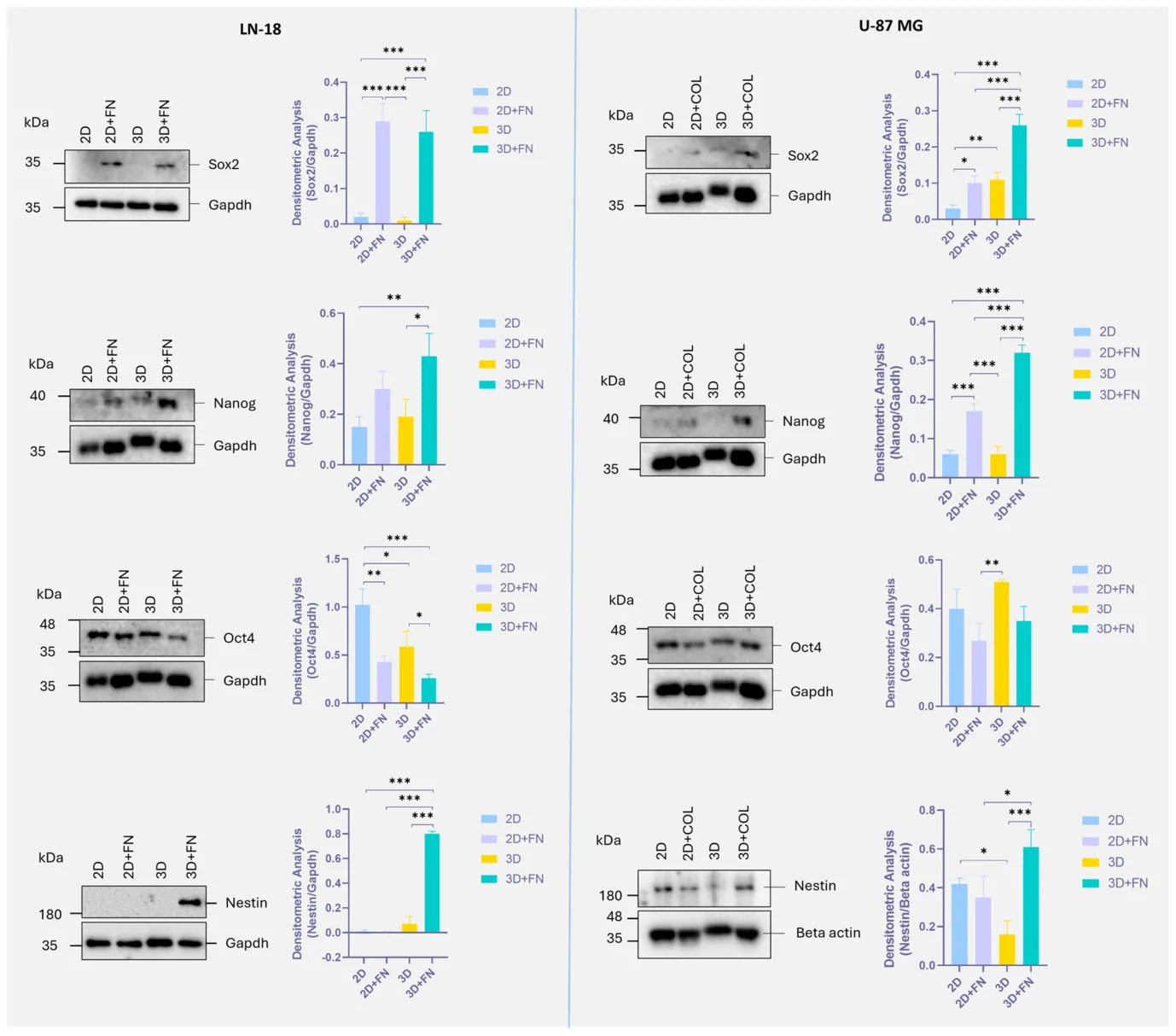
Expression of stemness-associated proteins in LN-18 and U-87 MG glioblastoma cells cultured under 2D and 3D conditions. Representative Western blot images and densitometric analyses of Sox2, Nanog, Oct4, and Nestin expression in LN-18 and U-87 MG glioblastoma cells cultured on conventional 2D surfaces and 3D ITO/PAN/C500 scaffolds for 7 days. LN-18 cultures were maintained in the presence or absence of fibronectin (FN), whereas U-87 MG cultures were maintained in the presence or absence of collagen IV (COL). Protein expression levels were normalized to GAPDH or β-actin as indicated. For U-87 MG cells, the β-actin loading control is the same as that shown for E-cadherin in Figure 1 because both proteins were detected sequentially on the same membrane following stripping and re-probing. Data are presented as mean ± SD from three independent experiments (*n* = 3). Statistical significance was determined using one-way ANOVA followed by Tukey’s multiple comparison test. *p* < 0.05 (*), *p* < 0.01 (**), and *p* < 0.001 (***). Original uncropped Western blot images are provided in Supplementary Figures S1 and S2.

Western blot analysis demonstrated substantial alterations in stemness-associated protein expression in response to ECM-functionalized 3D PAN/C500 scaffolds in both LN-18 and U-87 MG glioblastoma cells (Figure 4).

In LN-18 cells, Sox2 expression was significantly elevated in both the 2D+FN and 3D+FN groups compared with uncoated 2D cultures (*p* < 0.001), whereas minimal expression was detected in cells cultured on 3D scaffolds without fibronectin. Nanog expression followed a similar pattern, with the highest levels observed in the 3D+FN group, which showed significantly increased expression compared with both 2D and 3D cultures (*p* < 0.05–0.01). Nestin expression exhibited the most pronounced response, remaining nearly undetectable in 2D cultures while showing a marked increase in the 3D+FN group (*p* < 0.001). In contrast, Oct4 expression was significantly reduced under ECM-supported culture conditions (*p* < 0.001).

In U-87 MG cells, Sox2 expression increased progressively with ECM supplementation and 3D culture, reaching its highest level in the 3D+COL group. This increase was significant compared with all other experimental groups (*p* < 0.001). Similarly, Nanog expression was markedly elevated in the 3D+COL group and was significantly higher than in 2D, 2D+COL, and 3D cultures (*p* < 0.001). Nestin expression also increased substantially under 3D+COL conditions, exhibiting significantly higher levels than both 2D and 3D cultures without collagen (*p* < 0.05–0.001). Unlike the other stemness-associated markers, Oct4 expression displayed relatively modest changes among the experimental groups, although a significant increase was observed in 3D cultures compared with 2D+COL conditions (*p* < 0.01).

These findings indicate that ECM-functionalized 3D PAN/C500 scaffolds promote the expression of stemness-associated proteins, particularly Sox2, Nanog, and Nestin, in glioblastoma cells. The observed response was most pronounced in the ECM-coated 3D groups, suggesting that the combined effects of three-dimensional architecture and ECM cues promote stemness-associated molecular changes.

## 4. Discussion

The tumor microenvironment plays a critical role in regulating glioblastoma progression, invasion, and therapeutic resistance. In the present study, ECM-functionalized PAN/C500 scaffolds induced significant alterations in EMT-associated proteins, cytoskeletal organization, and stemness-associated molecular markers, indicating that microenvironmental cues profoundly influence glioblastoma cell behavior. The tumor microenvironment comprises extracellular matrix components, hypoxic niches, soluble signaling molecules, and surrounding stromal and neural cell populations, all of which contribute to tumor progression and phenotypic heterogeneity [5,33,34]. Consequently, the development of experimental models capable of reproducing these complex interactions has become a major focus of glioblastoma research. Recent studies have further emphasized that glioblastoma evolution is strongly influenced by intratumoral heterogeneity and dynamic interactions with the surrounding microenvironment, which collectively contribute to therapeutic resistance and disease progression [35,36].

Conventional 2D culture systems have been widely used for decades because of their simplicity and reproducibility. However, these systems fail to adequately mimic the spatial architecture, mechanical properties, and cell–ECM interactions encountered in vivo. Consequently, cellular responses observed in monolayer cultures often differ substantially from those observed in tumors, limiting their ability to predict clinically relevant therapeutic responses [21,37]. 3D culture systems have therefore emerged as more physiologically relevant alternatives that better recapitulate tumor architecture and microenvironmental signaling. In particular, electrospun nanofiber scaffolds have attracted considerable attention because their highly porous structure closely resembles native ECM organization and supports cell adhesion, migration, proliferation, and tissue-like cellular arrangements [38,39]. Consistent with this concept, spheroid and scaffold-based glioblastoma models have been shown to better recapitulate tumor architecture, oxygen gradients, cellular heterogeneity, and drug resistance patterns observed in vivo than conventional monolayer cultures [21,22,40].

Building upon our previous optimization studies, the present work focused on the biological characterization of the integrated ECM-functionalized PAN/C500 scaffold under three-dimensional culture conditions. Therefore, our objective was to evaluate the cellular responses elicited by the engineered scaffold as a whole rather than to dissect the individual contributions of its constituent materials. Using an optimized ECM-functionalized PAN/C500 scaffold, we demonstrate that ECM-derived microenvironmental cues profoundly influence glioblastoma cell phenotype under three-dimensional culture conditions. As the biological performance of the PAN/C500 platform has been characterized previously, cross-sectional H&E analyses demonstrated cellular infiltration by both LN-18 and U-87 MG cells into the nanofiber layer, indicating that cell–scaffold interactions extend beyond the scaffold surface. Through a process known as dynamic reciprocity, cells continuously interact with and remodel their surrounding matrix, while the ECM simultaneously influences gene expression, cytoskeletal organization, and cellular phenotype [41]. Sood et al. demonstrated that brain-derived ECM-containing 3D microenvironments support tumor-specific phenotypes and ECM-dependent transcriptional responses in glioblastoma cells, highlighting the importance of incorporating physiologically relevant ECM cues into in vitro brain tumor models [42]. Components such as collagen IV and fibronectin are abundant within the glioblastoma microenvironment and contribute to cell adhesion, migration, and mechanotransduction processes [7,8]. In the present study, we combined ECM functionalization with PAN/C500 nanofiber scaffolds to create a biomimetic platform that provides both structural support and physiologically relevant ECM signals. Our findings demonstrate that this microenvironment induces significant phenotypic adaptations in glioblastoma cells that are not observed under conventional culture conditions.

One of the most notable observations was the alteration of EMT-associated protein expression following culture on ECM-functionalized 3D scaffolds. Although glioblastoma is not a classical epithelial malignancy, EMT-associated molecular markers are widely used to evaluate cellular plasticity and mesenchymal-like adaptations in GBM. Therefore, the observed alterations in E-cadherin and N-cadherin should be interpreted as changes in EMT-associated molecular markers reflecting cellular plasticity rather than evidence of a canonical epithelial–mesenchymal transition. Vimentin expression was significantly increased in both LN-18 and U-87 MG cells under ECM-supported 3D conditions. In LN-18 cells, this increase was accompanied by a marked reduction in E-cadherin expression, whereas U-87 MG cells displayed elevated levels of both vimentin and N-cadherin. The observed changes do not indicate a canonical epithelial–mesenchymal transition but rather support EMT-associated molecular adaptations, particularly given the variable E-cadherin and N-cadherin responses observed between the two cell lines. Increased vimentin expression, together with cytoskeletal remodeling, suggests enhanced cellular plasticity and the acquisition of mesenchymal-like molecular features. Such intermediate or partial EMT-associated molecular states have increasingly been recognized in glioblastoma and are thought to promote tumor cell plasticity, invasiveness, and therapeutic resistance. The elevated vimentin expression observed in our study is particularly noteworthy, as vimentin is widely associated with invasive behavior, cytoskeletal flexibility, and poor prognosis across multiple malignancies, including gliomas.

The fluorescence imaging data further supported these findings. Live-cell vimentin imaging revealed increased fluorescence intensity and a more organized intermediate filament network in cells cultured on 3D scaffolds. Importantly, these observations were consistent with Western blot analyses, suggesting that the increased vimentin signal reflected genuine protein upregulation rather than differences in cell density alone. Intermediate filaments are essential regulators of cellular mechanics and migration, and vimentin reorganization has been associated with cellular plasticity and enhanced invasive behavior in glioblastoma. The more pronounced filamentous organization observed under 3D conditions, therefore, suggests that scaffold-mediated microenvironmental cues may promote a more physiologically relevant cytoskeletal organization.

In parallel with alterations in intermediate filaments, significant changes were also observed in actin cytoskeletal organization. Although total F-actin fluorescence did not differ significantly between 2D and 3D cultures, cell-normalized fluorescence intensity was significantly increased under 3D conditions in both cell lines. These findings indicate that 3D culture primarily influences actin organization rather than overall actin abundance. Actin remodeling is a critical component of cell migration, mechanosensing, and matrix interaction, and the formation of more organized actin networks under 3D conditions likely reflects enhanced engagement with the surrounding ECM. Such observations are consistent with previous reports demonstrating that ECM architecture can regulate cytoskeletal geometry and, in turn, influence gene expression through nuclear-cytoskeletal signaling pathways [42]. The simultaneous increase in vimentin expression and actin reorganization observed in our study suggests coordinated cytoskeletal remodeling in response to the biomimetic scaffold environment.

Beyond cytoskeletal alterations, one of the most important findings of the present study was the substantial modulation of stemness-associated proteins. Glioblastomas are increasingly recognized as hierarchically organized tumors containing subpopulations of glioblastoma stem-like cells, which are believed to contribute to tumor initiation, therapeutic resistance, recurrence, and disease progression [9,43]. These cells possess self-renewal capacity and exhibit molecular characteristics that overlap with embryonic and neural stem cells. Consequently, markers such as SOX2, NANOG, OCT4, and Nestin are frequently used to evaluate stem-like phenotypes in glioblastoma models.

SOX2 is one of the most extensively studied regulators of glioblastoma stemness and is essential for maintaining tumor-initiating cell populations. Gangemi et al. (2009) demonstrated that SOX2 silencing suppresses proliferation and tumorigenicity in glioblastoma stem cells, highlighting its central role in tumor maintenance [10]. Similarly, Lopez-Bertoni et al. (2022) reported that SOX2 promotes stemness and tumor propagation through epigenetic mechanisms [11]. In the present study, SOX2 expression was significantly elevated in ECM-supported 3D cultures, suggesting that the scaffold environment promotes stemness-associated molecular programs.

NANOG represents another critical regulator of pluripotency and self-renewal. Together with OCT4 and SOX2, NANOG forms a core transcriptional network responsible for maintaining embryonic stem cell identity [44,45,46]. In glioblastoma, NANOG has been linked to tumor stem cell maintenance and therapeutic resistance, while interactions with Hedgehog/GLI signaling further support malignant progression [16]. The increased NANOG expression observed in our ECM-functionalized 3D cultures, therefore, supports the notion that biomimetic microenvironments promote molecular adaptations associated with stemness.

Among all investigated markers, Nestin exhibited the most pronounced response, particularly in LN-18 cells cultured under 3D+FN conditions. Nestin is widely recognized as a marker of neural stem and progenitor cells and is frequently associated with aggressive glioma phenotypes [47]. Increased Nestin expression has previously been correlated with undifferentiated cellular states, enhanced tumor aggressiveness, and poor clinical outcome in glioma patients [48]. Consistent with these observations, the marked increase in Nestin expression observed in our study suggests that ECM-supported 3D culture conditions promote stemness-associated molecular changes within a biomimetic microenvironment.

In contrast to SOX2, NANOG, and Nestin, OCT4 displayed a more variable response depending on cell line and culture conditions. This finding is not entirely unexpected, as OCT4 expression in glioblastoma has been reported to be more heterogeneous than that of other stemness-associated markers and may depend on specific molecular subtypes or differentiation states [49,50]. Nevertheless, the overall pattern observed in this study supports the conclusion that the 3D scaffold environment promotes molecular adaptations associated with stemness.

The divergent responses of LN-18 and U-87 MG cells to the ECM-functionalized PAN/C500 platform may reflect their distinct intrinsic molecular backgrounds rather than a uniform scaffold-induced response. LN-18 cells are commonly characterized as PTEN-proficient, TP53-mutant, and MGMT-active, whereas U-87 MG cells are PTEN-deficient, TP53-wild-type, and generally exhibit low or absent MGMT activity [51]. These molecular differences are biologically relevant because PTEN/PI3K/AKT- and p53-associated signaling influence cell adhesion, cytoskeletal organization, differentiation, survival, and responses to microenvironmental stimuli [52]. Consistent with this concept, PTEN-proficient LN-18 and PTEN-deficient U-87 MG cells have been reported to exhibit distinct cellular responses under comparable experimental conditions, supporting the notion that their genetic backgrounds shape their adaptation to extracellular cues [53]. Whole-exome and transcriptomic analyses have further demonstrated substantial molecular heterogeneity among established glioblastoma cell lines, including differences in TP53, PTEN, EGFR, NF1, and genes involved in cell adhesion and differentiation [54]. Moreover, U-87 MG cells have been reported to display greater invasive capacity than LN-18 cells, highlighting functional differences that extend beyond their genetic profiles [55]. Therefore, the cell line-dependent changes in EMT-associated and stemness-associated markers observed in the present study may represent distinct adaptive responses to the same biomimetic microenvironment rather than inconsistent scaffold effects. This observation highlights the importance of evaluating biomimetic culture platforms using molecularly distinct glioblastoma models and supports the utility of the ECM-functionalized PAN/C500 platform for investigating tumor heterogeneity and cell line-dependent biological responses in vitro.

Our findings extend previous observations obtained with electrospun glioblastoma models. Ma et al. (2016) demonstrated that three-dimensional electrospun scaffolds in combination with ECM components promote glioma stemness through enhanced ECM signaling and clonogenic potential. In agreement with these findings, our study also supports the importance of ECM-supported 3D culture in promoting stemness-associated molecular changes. However, rather than focusing on clonogenicity and integrin-mediated signaling, we provide a broader molecular characterization by simultaneously evaluating stemness-associated proteins together with EMT-associated proteins and cytoskeletal remodeling in two glioblastoma cell lines cultured on a previously optimized ECM-functionalized PAN/C500 scaffold [26]. Similarly, Saleh et al. demonstrated that laminin-functionalized aligned nanofiber scaffolds preserve the invasive plasticity of glioblastoma stem-like cells and modulate migration-associated pathways by recapitulating the structural and biochemical characteristics of the brain extracellular matrix. While their study primarily focused on migration behavior and invasion-related signaling, our work extends the biological characterization of ECM-functionalized electrospun scaffolds by demonstrating coordinated changes in cytoskeletal organization, EMT-associated proteins, and stemness-associated molecular markers under biomimetic 3D culture conditions [56]. Unlike previously reported electrospun glioblastoma models, which have primarily focused on scaffold fabrication, ECM incorporation, or the expression of stemness-associated markers, the present study provides a comprehensive molecular characterization of glioblastoma cells cultured on a previously optimized ECM-functionalized PAN/C500 scaffold. By integrating analyses of EMT-associated proteins, live-cell cytoskeletal organization, and stemness-associated molecular markers in two glioblastoma cell lines, our study expands the biological validation of this scaffold platform and provides further insight into the phenotypic adaptations induced by a biomimetic three-dimensional microenvironment.

The results presented here indicate that ECM-functionalized PAN/C500 scaffolds provide a biologically relevant microenvironment that induces coordinated molecular and structural changes in glioblastoma cells. The observed increases in vimentin, SOX2, NANOG, and Nestin expression, together with extensive cytoskeletal remodeling, suggest that cells cultured in this 3D system acquire phenotypic characteristics that more closely resemble those observed in vivo. These findings support the utility of ECM-functionalized PAN/C500 nanofiber scaffolds as biologically relevant in vitro glioblastoma models for studying tumor biology and investigating molecular mechanisms associated with cellular plasticity and stemness. The coordinated modulation of cytoskeletal organization, EMT-associated proteins, and stemness-related markers further highlights the importance of ECM-mediated microenvironmental cues in shaping glioblastoma cell behavior. Although the molecular mechanisms linking ECM-derived signals to the observed phenotypic adaptations were not investigated in the present study, mechanotransduction pathways are likely to contribute to these responses and will be explored in our future studies. Complementary to this, future work should incorporate functional migration and invasion assays to determine whether the molecular and cytoskeletal adaptations observed here translate into corresponding changes in glioblastoma cell behavior. Such investigations will require specifically adapted experimental configurations, as the current ITO-supported nanofiber platform was designed for molecular and imaging-based characterization rather than conventional transwell or spheroid invasion assays. In addition, further functional studies will be important to determine whether the stemness-associated molecular changes observed in the present study translate into corresponding functional stem cell properties. Furthermore, establishing the functional relevance of these molecular adaptations will also support future applications of this platform in therapeutic response studies.

## 5. Conclusions

The present study demonstrates that ECM-functionalized PAN/C500 nanofiber scaffolds significantly influence glioblastoma cell behavior by promoting coordinated molecular and structural adaptations under 3D culture conditions. Compared with conventional 2D cultures, glioblastoma cells grown on 3D scaffolds exhibited pronounced alterations in EMT-associated protein expression, enhanced vimentin organization, cytoskeletal reorganization, and increased expression of stemness-related markers, including SOX2, NANOG, and Nestin. These findings suggest that the combined effects of 3D architecture and ECM signaling promote molecular and structural adaptations associated with cellular plasticity and the expression of stemness-associated markers. Furthermore, the observed differences between 2D and 3D cultures highlight the limitations of conventional monolayer systems in reproducing the complex microenvironmental cues present in glioblastoma. Our results support the use of ECM-functionalized PAN/C500 scaffolds as a biologically relevant in vitro glioblastoma model that more closely mimics tumor-associated cellular responses. This platform provides a biologically relevant in vitro glioblastoma model that more closely mimics tumor-associated microenvironmental cues and may facilitate future investigations into glioblastoma biology, cellular plasticity, stemness-associated molecular changes, and therapeutic responses.

## Data Availability

The original contributions presented in this study are included in the article/Supplementary Material. Further inquiries can be directed to the corresponding author.

## Supplementary Materials

The following supporting information can be downloaded at https://www.mdpi.com/article/10.3390/biom16081077/s1, Supplementary Figure S1: Uncropped Western blot images and corresponding processed blots of EMT- and stemness-related proteins in LN18 glioblastoma cells. Supplementary Figure S2: Uncropped Western blot images and corresponding processed blots of EMT- and stemness-related proteins in U87 glioblastoma cells.
