# Supplementary material for: Three-Dimensional ECM-Functionalized PAN/C500 Nanofiber Scaffolds Induce Cytoskeletal Remodeling and Stemness-Associated Molecular Changes in Glioblastoma Cells
Source: Biomolecules. 2026 Jul 23;16(8):1077. doi: 10.3390/biom16081077 (PMC13510142; doi:10.3390/biom16081077)
Supplement: Supplementary file 1 [file biomolecules-16-01077-s001.zip › biomolecules-4422210-supplementary.pdf]

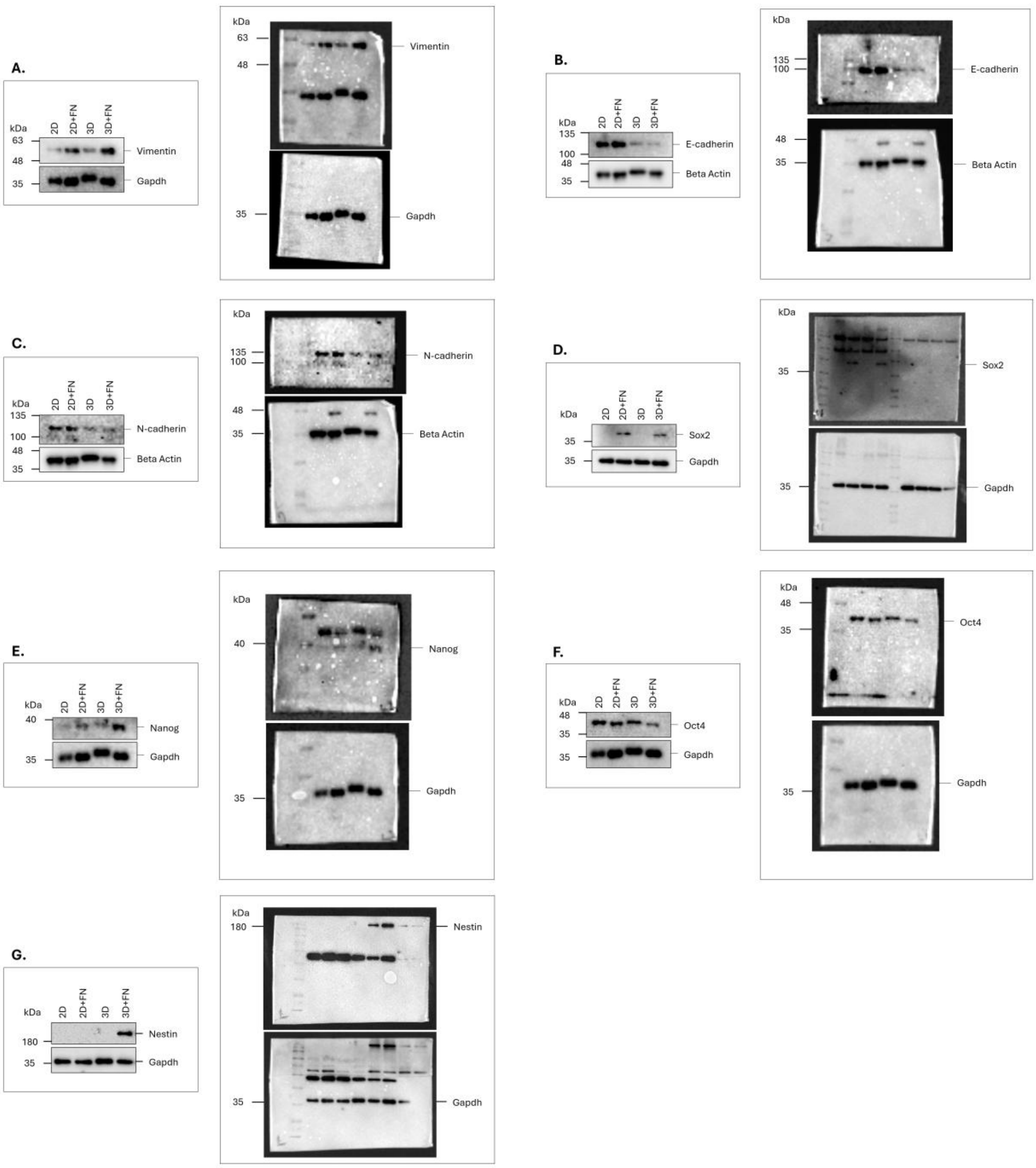

Supplementary Figure S1. Uncropped Western blot images and corresponding processed blots of EMT- and stemness-related proteins in LN18 glioblastoma cells. Representative processed (cropped) Western blot images (left) together with their corresponding full-length, uncropped membrane images (right) showing the expression of **(A)** Vimentin, **(B)** E-cadherin, **(C)** N-cadherin, **(D)** Sox2, **(E)** Nanog, **(F)** Oct4, and **(G)** Nestin in LN18 glioblastoma cells under the indicated experimental conditions (2D, 2D+FN, 3D, and 3D+FN). GAPDH or β-actin served as the loading control, as indicated for each target protein. Molecular weight markers (kDa) are shown on the left side of each membrane.

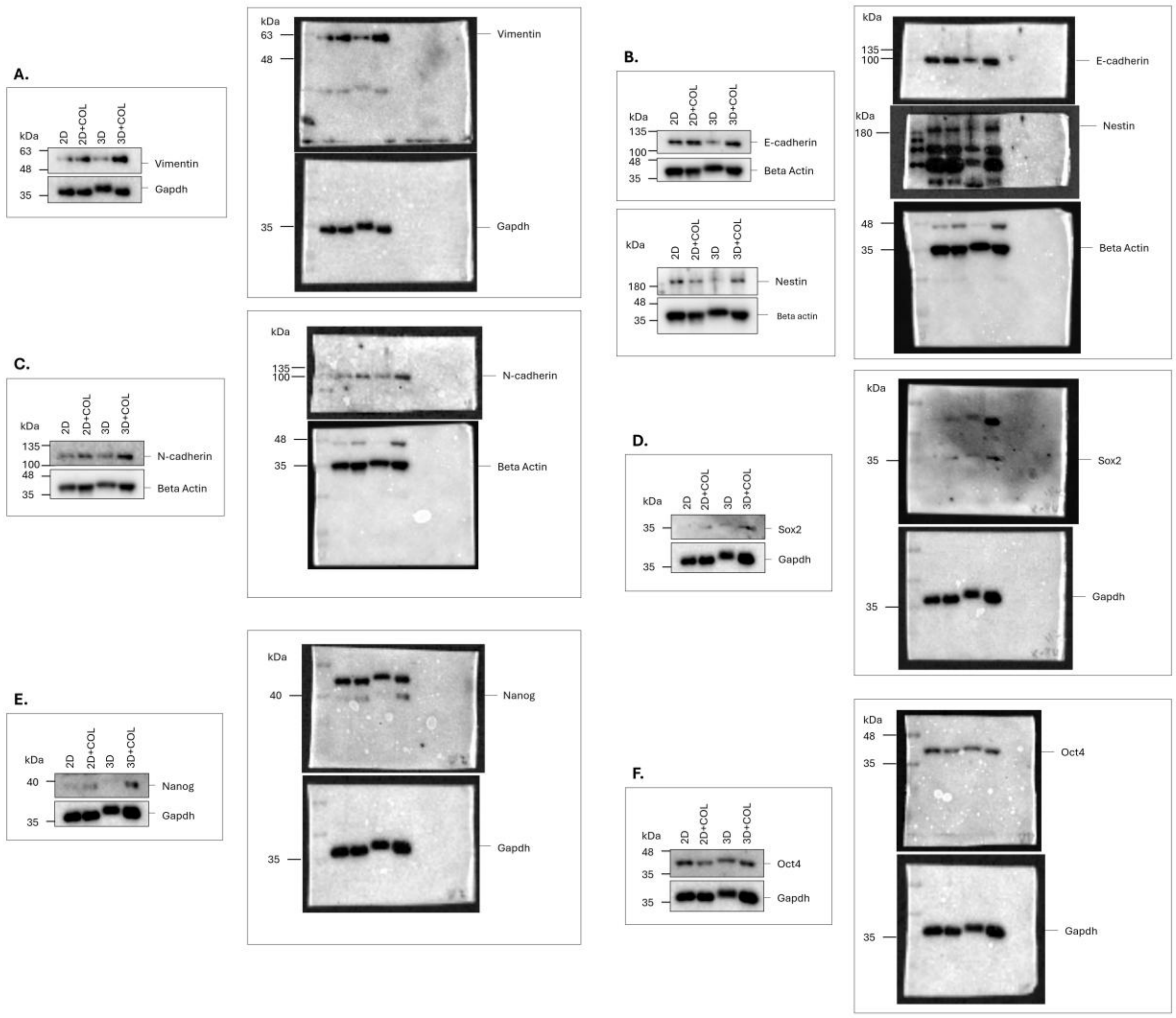

Supplementary Figure S2. Uncropped Western blot images and corresponding processed blots of EMT- and stemness-related proteins in U87 glioblastoma cells. Representative processed (cropped) Western blot images (left) together with their corresponding full-length, uncropped membrane images (right) showing the expression of **(A)** Vimentin, **(B)** E-cadherin and Nestin, (**C)** N-cadherin, **(D)** Sox2, **(E)** Nanog, and **(F)** Oct4 in U87 glioblastoma cells cultured under the indicated experimental conditions (2D, 2D+COL, 3D, and 3D+COL). GAPDH or β-actin served as the loading control, as indicated for each target protein. Molecular weight markers (kDa) are shown on the left side of each membrane.

Note: *In U87-MG cells, the β-actin loading control presented for Nestin (Figure 4) is the same as that shown for E-cadherin (Figure 1), because both target proteins were analyzed sequentially on the same membrane after stripping and re-probing.*
